# Large language models in nephrology: applications and challenges in chronic kidney disease management

**DOI:** 10.1080/0886022X.2025.2555686

**Published:** 2025-09-07

**Authors:** Yongzheng Hu, Jianping Liu, Wei Jiang

**Affiliations:** ^a^ Department of Nephrology, The Affiliated Hospital of Qingdao University, Qingdao, China; ^b^ Department of medical administration, University of Health and Rehabilitation Sciences (Qingdao Municipal Hospital), Qingdao, China

**Keywords:** Large language models (LLMs), artificial intelligence (AI), CKD, clinical decision support, digital health

## Abstract

Large language models (LLMs) represent a transformative advance in artificial intelligence, with growing potential to impact chronic kidney disease (CKD) management. CKD is a complex, highly prevalent condition requiring multifaceted care and substantial patient engagement. Recent developments in LLMs—including conversational AI, multimodal integration, and autonomous agents—offer novel opportunities to enhance patient education, streamline clinical documentation, and support decision-making across nephrology practice. Early reports suggest that LLMs can improve health literacy, facilitate adherence to complex treatment regimens, and reduce administrative burdens for clinicians. However, the rapid deployment of these technologies raises important challenges, including patient privacy, data security, model accuracy, algorithmic bias, and ethical accountability. Moreover, real-world evidence supporting the safety and effectiveness of LLMs in nephrology remains limited. Addressing these challenges will require rigorous validation, robust regulatory frameworks, and ongoing collaboration between clinicians, AI developers, and patients. As LLMs continue to evolve, future efforts should focus on the development of nephrology-specific models, prospective clinical trials, and strategies to ensure equitable and transparent implementation. If appropriately integrated, LLMs have the potential to reshape the landscape of CKD care and education, improving outcomes for patients and supporting the nephrology workforce in an era of increasing complexity.

## Graphic Abstract

**Figure:**
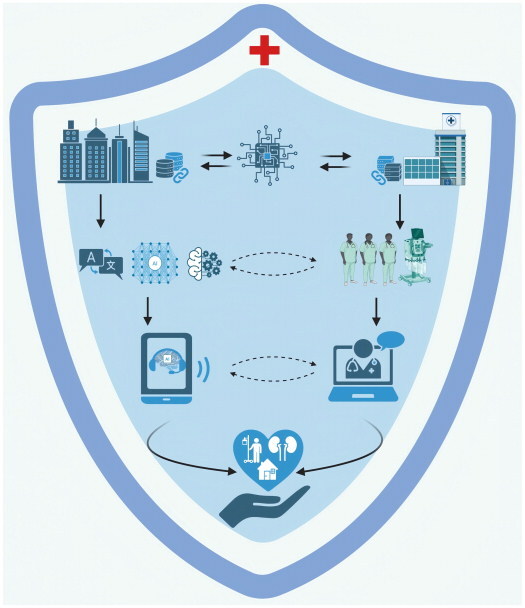

## 1. Introduction

Chronic kidney disease (CKD) imposes a growing global burden, affecting more than 10% of the world’s population—more than 800 million individuals. The disease is often accompanied by comorbidities such as hypertension, diabetes, and cardiovascular disease, so complex patient management that encompasses frequent monitoring, polypharmacy, and tailored lifestyle modifications is needed [1]. Despite the lifesaving potential of renal replacement therapies such as dialysis and transplantation, their high cost and limited availability—especially in lower- and middle-income countries—highlight significant limitations in current standards of care. Moreover, CKD-associated mortality remains high, and patient awareness is low, with up to 90% unaware of their condition. In this context, there is an urgent need for advanced supportive technologies [2].

Large language models (LLMs) represent an advanced category of artificial intelligence technology capable of generating human-like text on diverse datasets on the basis of extensive training [3]. The introduction of models such as ChatGPT has marked a significant turning point, demonstrating unprecedented capability in understanding and generating complex dialog. Notably, these technologies have swiftly progressed, with newer models achieving high proficiency levels, including strong performance on standardized medical examinations [4]. Although the technology cannot replace the care provided by human or healthcare professionals, given their rapid advancement and versatile capabilities, LLMs hold substantial promise for transforming healthcare practices, patient management, and medical education, although their integration requires careful evaluation of associated benefits and potential limitations.

Nephrology, a field that involves managing complex chronic diseases (e.g., CKD, dialysis care, and transplantation), stands to both benefit and face challenges from LLM integration [5]. This review examines the impact of LLMs on nephrology clinical practice. We focus on (1) current and emerging applications in nephrology, including patient engagement, workflow optimization, and medical training; (2) recent technical advances (2022–2025), such as voice interfaces, multimodal capabilities, and autonomous agents that can enhance nephrology care; (3) a comparative analysis of LLM-based virtual assistants versus traditional internet-based ‘online doctor’ models; (4) ethical, security, and regulatory challenges in deploying LLMs in nephrology; and (5) future directions for research and clinical adoption. The aim is to provide a clinically oriented, up-to-date overview suitable for the digital health community.

## 2. Applications of LLMs in nephrology

LLMs have a range of capabilities that can be leveraged in nephrology practice, from interactive patient education to assisting clinicians with data management and decision support. The key application domains include patient engagement, clinical workflow improvements, and medical education.

### 2.1. Enhancing patient engagement and self-management

Effective self-management of chronic illness is a complex, interactive process involving not only patients but also their families and supportive technologies. According to Grey and colleagues, optimal chronic disease management involves addressing illness-specific tasks, activating critical resources—including emotional coping strategies, healthcare services, and family support—and integrating management activities meaningfully into daily routines. Within nephrology, due to the complexity of conditions such as CKD, significant patient involvement, rigorous adherence to treatment regimens, and active engagement from family members who provide essential support are needed. Integrating LLMs into these self-management frameworks offers innovative opportunities by facilitating real-time, personalized patient education, improving communication between patients and healthcare providers, and reinforcing family support mechanisms [6,7].

Effective patient education and engagement are cornerstones of nephrology, in which patients must manage complex regimens (diet, medications, and dialysis schedules) [8]. LLM-based chatbots offer a new way to support patients between visits by answering questions and reinforcing medical advice in an accessible conversational format [9]. For example, an LLM-powered virtual assistant could explain laboratory results or dialysis procedures to a patient in layperson terms, helping demystify their condition. These assistants are recognized as promising applications of ChatGPT in medicine, assisting patients in managing their health through conversational interactions [10]. They can provide medication reminders, dosage instructions, and warnings about potential side effects or drug interactions, which is particularly valuable for kidney patients with polypharmacy. In one report on artificial intelligence (AI) for chronic disease self-management, an LLM-based agent was able to reliably converse with patients to collect health information across a range of diseases, highlighting its versatility [11].

In addition to conveying information, LLMs can engage patients in two-way dialog that may improve health literacy and empowerment [12]. A nephrology chatbot could quiz patients about their dietary choices (‘Can you eat this food on a low-potassium diet?’) and provide corrective feedback or help them log symptoms and fluid intake between dialysis sessions. This interactive coaching can increase adherence to care plans (e.g., fluid restriction in dialysis) and serve as a triage tool. For example, if a patient describes worrying symptoms (such as new-onset shortness of breath or edema), the LLM could recognize danger signs and prompt the patient to seek immediate medical attention or notify clinicians. While such use is experimental, it illustrates how LLMs might personalize care beyond the clinic. Importantly, any patient-facing AI must provide safe and accurate information and know its limits; current models tend to offer generic advice and could miss subtle nuances in an individual’s case. Thus, in practice, these systems require oversight and careful tuning of each patient’s context. Early experiences in other fields suggest that patients often find chatbot explanations articulate and encompassing but not yet perfectly tailored [13]. In nephrology, ongoing pilot studies and patient feedback will be critical to refine LLM communication for this sensitive domain.

### 2.2. Optimizing the clinical workflow and decision support

Nephrologists and renal care teams face substantial documentation and data synthesis burdens [14]. Integrating LLMs into nephrology practice presents significant opportunities for streamlining clinical documentation and enhancing patient data management. Through advanced voice-to-text and natural language processing technologies, LLMs can efficiently transcribe and summarize patient consultations in real time, automatically generating structured and comprehensive electronic health record (EHR) entries [15]. For example, during dialysis rounds or clinic visits, key clinical details such as patient symptoms, medication adjustments, laboratory results, and treatment plans can be rapidly captured and organized, significantly reducing administrative burdens and minimizing documentation errors. By automating these traditional time-consuming tasks, nephrologists can dedicate more time to direct patient interactions, thereby improving clinical efficiency and patient care quality.

Moreover, LLMs have demonstrated considerable promise in providing real-time clinical decision support tailored specifically to nephrology contexts. Leveraging patient-specific data—such as laboratory values, medication profiles, and comorbid conditions—in conjunction with the latest nephrology guidelines, these models can deliver personalized, evidence-based treatment recommendations [16]. For example, AI-driven decision-support systems have been effectively utilized to predict acute kidney injury (AKI), enabling clinicians to initiate preventive measures well in advance. Furthermore, in outpatient settings, LLMs can aid in accurately triaging patient communications, efficiently categorizing inquiries according to urgency and facilitating timely referrals to subspecialists. Such capabilities not only enhance clinical decision-making but also ensure optimal resource allocation and patient safety within nephrology practices.

Finally, the integration of LLMs extends beyond direct patient care to broader administrative and continuous learning functions [17]. These models can autonomously manage routine administrative responsibilities, including scheduling dialysis sessions, coordinating medication refills, and organizing follow-up appointments, substantially alleviating workload pressures on clinical staff. Additionally, LLMs offer adaptive capabilities through continuous learning mechanisms, regularly updating their knowledge base with the latest nephrology research findings, guidelines, and clinical protocols [18]. This ongoing adaptability ensures that nephrologists receive the most current information and recommendations, enabling them to consistently deliver high-quality, evidence-informed patient care in a rapidly evolving medical landscape.

### 2.3. Medical education and training

The integration of LLMs into nephrology education and training has transformative potential, particularly in enhancing learning experiences and clinical decision-making. Recent studies have demonstrated that LLMs can answer nephrology-specific questions with a high level of accuracy, with performance metrics indicating significant improvements when advanced prompting techniques such as chain-of-thought reasoning are employed [19]. In addition to their question-answering capabilities, LLMs have been instrumental in simplifying complex nephrology concepts for both trainees and patients. By generating patient-friendly explanations and translating intricate medical information into more accessible language, these models facilitate better understanding and engagement [20]. Moreover, with the integration of retrieval-augmented generation (RAG) techniques, LLMs can access up-to-date medical guidelines and research, ensuring that the information provided aligns with current best practices [21]. They can condense textbook chapters, research articles, or clinical practice guidelines into concise explanations. For example, a nephrology fellow could ask an LLM to summarize the latest KDIGO guidelines on blood pressure management in CKD patients and use that overview as a study aid or memory refresher before the clinic. The model can highlight key points (target blood pressure, recommended medications, etc.), accelerating knowledge acquisition. This dynamic access to information is particularly beneficial in fields such as nephrology, where guidelines and treatment protocols are continually evolving.

In clinical training settings, LLMs serve as valuable tools for simulating patient interactions and clinical scenarios. By presenting trainees with diverse case studies and guiding them through diagnostic and treatment processes, these models offer a risk-free environment to develop and refine their clinical skills [22]. Such applications not only enhance the learning experience but also prepare trainees for real-world clinical decision-making. Furthermore, the adaptability of LLMs allows for the customization of training modules to address specific educational needs within nephrology, fostering a more personalized learning approach [23].

While the integration of LLMs into nephrology education presents numerous advantages, potential challenges must be. Concerns regarding the accuracy of generated information, the potential for AI ‘hallucinations,’ and the need for continuous validation against clinical standards must be carefully managed [24]. Ensuring that LLMs are used as adjunct tools—complementing rather than replacing traditional educational methods—is crucial for maintaining the integrity and effectiveness of medical training programs.

## 3. Recent technical advances (2022–2025) and their potential in nephrology

The capabilities of LLMs have expanded considerably in recent years, enabling new modes of interaction and functionality that could further enhance their usefulness in clinical care. Three important technical advances are as follows: voice interaction, multimodal input/output, and autonomous AI ‘agent’ behavior [25,26]. Each of these advances can be viewed through the lens of how they might contribute to nephrology practice.

### 3.1. Voice interaction and conversational AI

LLMs have opened new avenues for enhancing patient care and clinical efficiency [27]. One significant advancement is the development of voice-based conversational AI applications tailored to assisting patients in managing complex treatment regimens. For example, a randomized clinical trial demonstrated that a voice-based conversational AI application significantly improved insulin dose optimization among patients with type 2 diabetes, leading to better glycemic control and medication adherence [28]. This success highlights the potential for similar applications in nephrology, such as assisting patients in managing medication schedules and dietary restrictions crucial for CKD management.

Moreover, LLM-equipped voice assistants can facilitate seamless communication between healthcare providers and patients who speak different languages. By leveraging advanced natural language processing capabilities, these AI-driven tools can provide real-time translation and interpretation services, ensuring that language barriers do not impede the delivery of quality care [29]. Additionally, the advanced voice mode enables more natural and empathetic communication with patients. This is particularly beneficial in nephrology, where complex treatment regimens require clear and compassionate explanations. The model’s ability to detect and respond to nonverbal cues, such as tone and speaking pace, can enhance patient comfort and adherence to treatment protocols [30].

Despite the significant potential of voice interaction and conversational AI to enhance patient engagement and clinical workflow, it is important to acknowledge specific limitations, particularly among patients with hearing impairments—a condition frequently observed in CKD populations. For patients experiencing hearing difficulties, reliance on voice-based interfaces might not only diminish the efficacy of communication but also exacerbate existing healthcare disparities. To mitigate these challenges, multimodal AI approaches that integrate visual and textual communication modes alongside auditory interaction should be prioritized. For example, LLM-based systems can provide simultaneous visual captions, real-time text summaries, or alternative communication interfaces tailored specifically for those with impaired hearing. Ensuring the development of accessible technologies that address these limitations is essential for the equitable and inclusive implementation of conversational AI solutions in nephrology practice [31,32].

On the other hand, the integration of voice recognition technology into electronic health record (EHR) systems has streamlined clinical documentation processes. Nephrologists can utilize voice commands to input patient data, review medical histories, and update treatment plans efficiently [33]. This reduces the administrative burden on clinicians, minimizes the risk of documentation errors, and allows more time for direct patient care. By addressing language barriers, enhancing patient engagement, and streamlining clinical workflows, these technological advancements hold the promise of significantly improving outcomes for patients with kidney diseases.

### 3.2. Multimodal capabilities

Current LLMs are no longer confined to text; they can accept and produce multiple data modalities (images, audio, and structured data) in addition to text [28]. Compared with previous models that rely on separate systems for different modalities, this integration enables more seamless and efficient interactions. In nephrology, these multimodal capabilities can be harnessed to increase diagnostic accuracy and patient education. For example, the ability of LLMs to process and interpret medical images alongside textual data allows for more comprehensive analysis of renal ultrasounds or biopsy slides. By combining visual data with patient history and laboratory results, they can assist clinicians in identifying patterns indicative of specific kidney pathologies.

Moreover, GPT-4o’s native image generation capabilities surpass those of previous models such as DALL·E 3, offering more accurate and contextually relevant visual outputs [34]. This advancement facilitates the creation of detailed educational materials, such as annotated diagrams of the nephron or visual explanations of dialysis procedures, tailored to individual patient needs. These personalized educational tools can improve patients’ understanding of and engagement in their treatment plans. The accuracy of image interpretation by AI models still requires rigorous validation, and the integration of such technologies into existing clinical workflows must be approached cautiously [35].

### 3.3. Autonomous AI agents

Perhaps the most radical advance in this regard is the emergence of AI agents that can operate autonomously by breaking down tasks and using tools, with LLMs as the reasoning engine [36]. These agents, which are characterized by their ability to perform tasks with minimal human intervention, are increasingly being explored for their potential to enhance various aspects of nephrology practice. One promising application is in the automation of administrative tasks within nephrology clinics. For example, AI agents can be designed to handle routine workflows such as scheduling patient appointments, managing laboratory test orders, and preparing dialysis reports. By automating these processes, clinicians can allocate more time to direct patient care, potentially improving overall efficiency and reducing the likelihood of administrative errors. In the realm of patient monitoring, autonomous AI agents can be used to continuously analyze data from wearable devices and electronic health records [37]. Such systems can detect early signs of complications, such as rising blood pressure or missed dialysis sessions, and proactively alert healthcare providers. This continuous monitoring facilitates timely interventions, which are crucial in managing CKD and preventing acute exacerbations.

Furthermore, the integration of AI agents into clinical decision support systems holds promise for enhancing diagnostic accuracy and treatment planning [38].

## 4. LLM-based virtual assistants vs. traditional online doctor models

The emergence of large language model (LLM)-based virtual assistants has stimulated significant interest in their comparison with traditional online doctor–patient interaction modalities, including telemedicine consultations with human clinicians and digital platforms such as symptom checkers and medical search engines [39]. LLM-based assistants offer unique advantages in scalability and operational consistency, delivering immediate, around-the-clock responses to patient inquiries without the limitations of clinician fatigue or scheduling constraints. Recent studies demonstrate that these virtual assistants can provide clearer and more guideline-concordant answers to frequently asked medical questions than conventional search-based tools, thus supporting patient needs with enhanced reliability [40]. Notably, LLMs maintain a consistent professional tone, and emerging evidence suggests that AI-generated responses may be perceived as more empathetic in online health forums than those provided by human physicians [41]. These findings highlight the potential utility of LLMs as adjuncts to patient counseling, particularly in delivering repetitive or routine health education that may be constrained by clinician workload. Furthermore, the capacity of LLMs for rapid and uniform updates with the latest medical evidence may enable the provision of current, standardized recommendations across large patient populations, provided the underlying models are appropriately maintained and validated [42].

By contrast, human physicians contribute indispensable strengths to virtual consultations, notably through clinical judgment, dynamic problem-solving, and the capacity to individualize care. Clinicians are trained to interpret subtle verbal and non-verbal cues, elicit critical contextual information, and iteratively adjust their clinical reasoning based on patient interactions—skills that are particularly vital in nephrology, where early recognition of nuanced symptoms may prevent serious complications. For instance, a nephrologist may detect signs of fluid overload through a patient’s narrative or demeanor and initiate timely intervention. Current LLMs, however, are inherently limited to processing only the information explicitly provided, and may overlook contextual factors or unarticulated concerns unless specifically prompted [43]. While advances in multimodal AI now permit the analysis of both text and images, these models have yet to replicate the nuanced synthesis performed by clinicians during integrated physical and diagnostic assessments. Tasks such as correlating a renal ultrasound with bedside findings, or visually inspecting a dialysis access site for infection, remain firmly within the clinician’s domain. Additionally, the presence of legal and ethical accountability distinguishes medical professionals from AI: licensed physicians are held to established standards of care and bear direct responsibility for clinical decisions, whereas LLMs currently operate without intrinsic accountability. This distinction underpins patient trust, as individuals may rely more readily on the guidance of a dedicated physician, assured by professional oversight and ethical obligations [44].

Another critical consideration in virtual healthcare is the provenance and traceability of medical information. Human clinicians, whether in traditional or telemedicine settings, can readily articulate their clinical reasoning, cite relevant guidelines, and draw upon personal experience to justify recommendations—thereby fostering transparency and patient trust. By contrast, standard LLMs typically do not disclose their sources, which poses challenges for verifying the accuracy and credibility of AI-generated medical advice. Recent comparative studies illustrate this gap: for example, while some advanced AI platforms, such as Microsoft’s Bing AI, are capable of providing source references for their responses, most general-purpose LLMs—including widely used models—do not, thereby affecting users’ confidence in the advice received [45]. Hybrid systems that combine the fluency of AI with explicit source citation may offer a more robust solution, enhancing traceability and clinical utility in future applications. The differences between LLM virtual assistants and traditional online doctor consultations are summarized in Table 1.

**Table 1.** Comparison of LLM virtual assistants with traditional online doctor consultations (human providers).

Aspect	LLM virtual assistant	Online human doctor (Telemedicine/consult)
Availability	24/7 instant access; no waiting times.	Limited to working hours/appointment times; possible delays for responses.
Consistency	Uniform quality and tone; does not tire or vary by time of day.	May vary with clinician’s workload, fatigue, or individual style.
Knowledge base	Vast training on general medical literature; can provide comprehensive background information.	Deep specialized expertise and experiential knowledge, but scope is limited to clinician’s training and memory.
Personalization	Lacks inherent patient-specific context; gives generic advice unless integrated with patient data.	Tailors advice to the individual’s history, nuances, and lab results as known to the doctor.
Empathy & rapport	Simulates empathy with polite, reassuring language; no genuine emotion, but often perceived as empathetic.	Genuine empathy and emotional support; can build trust through real human connection over time.
Clinical reasoning & judgment	Follows learned patterns; may miss unusual presentations or multifactorial nuances without explicit input.	Applies clinical reasoning to synthesize history, exam, and investigations; can handle ambiguity and ask clarifying questions proactively.
Actions & interventions	Provides advice in text (or speech) only; cannot perform physical exams, order tests, or prescribe treatments.	Can physically examine (if video or later in person), order appropriate tests, prescribe medications, and intervene directly.
Accuracy & safety	Prone to the phenomenon of ‘hallucination’ – confidently giving incorrect or fictitious information. No built-in error checking; relies on user to discern accuracy.	Advice bounded by clinician’s expertise and guidelines; far fewer outright fabrications. However, humans can err in diagnosis or advice, too.
Accountability	No legal accountability; considered a tool. Errors or harm from AI advice currently fall into a grey zone legally.	Licensed and legally accountable for advice given; subject to malpractice if negligence occurs.
Transparency	Provides answers without citing sources (unless specially designed to do so); patient cannot easily verify origin of information.	Can cite specific clinical guidelines, evidence, or personal clinical experience to justify recommendations, enhancing trust.
Privacy	May require sharing sensitive data with a third-party AI platform; potential HIPAA/GDPR compliance issues if not locally hosted.	Bound by doctor–patient confidentiality; uses secure communication channels as per healthcare regulations.
Cost	Low marginal cost per interaction; one AI can serve millions of users once developed. Typically, offered free or *via* subscription.	Significant cost per consultation (professional fees); not scalable to unlimited patients due to human resource limits. Insurance or out-of-pocket payment required for each visit.

Given the complementary strengths of AI and human clinicians, many experts now advocate for hybrid care models that leverage both. Rather than framing AI as a substitute for the physician, the optimal approach is collaborative: LLMs offer unmatched availability, consistency, and scalability, while human doctors provide critical contextualization, empathy, and the authority to enact medical interventions [46]. In practice, this synergy can manifest in various ways—for instance, LLMs may draft patient education materials or summarize routine consultations for clinician review, allowing physicians to focus on more complex or nuanced aspects of care. Some health systems have already begun piloting workflows in which AI-generated draft responses to patient inquiries are vetted and finalized by clinicians. In nephrology, such a division of labor could involve AI handling standard CKD education and administrative tasks, with escalation to a nephrologist when medical judgment or direct intervention is required. Ultimately, integrating the strengths of LLMs with those of clinicians—while mitigating the respective limitations—has the potential to maximize efficiency, maintain high standards of care, and improve patient outcomes.

## 5. Ethical, security, and regulatory challenges

**Figure 1.**
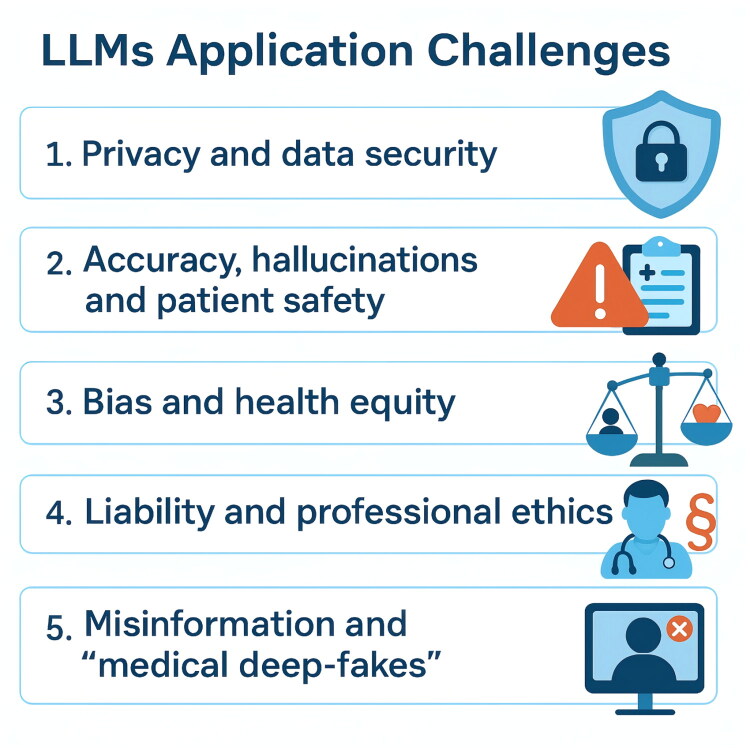
Key challenges in applying large language models (LLMs) in healthcare. This infographic summarizes the core challenges that must be addressed to ensure the safe and effective integration of LLMs into healthcare and nephrology practice.

The deployment of LLMs in nephrology (and healthcare at large) raises numerous ethical and security considerations. These range from patient privacy and data protection to the accuracy and safety of AI-provided advice to broader issues of bias, equity, and professional responsibility [47]. Addressing these challenges (shown in Figure 1) is paramount before LLMs can be trusted in routine clinical use.

### 5.1. Privacy and data security

LLMs achieve clinical relevance only if they can access patient information (medical history, labs, etc.), but this creates the risk of exposing sensitive data. Integrating an LLM with electronic health records or allowing it to handle protected health information must comply with health privacy laws such as the HIPAA in the U.S. and the GDPR in Europe [48]. A major concern is that many powerful LLMs are hosted on external servers (‘the cloud’), meaning that the data input to them could leave the hospital’s secure environment. In nephrology, if a physician asks an online AI about a specific patient’s case details, that could inadvertently transmit identifiable health data to a third party [49]. Strict safeguards and probably on-premises or encrypted implementations of LLMs will be needed for clinical use. Hospitals and AI vendors are exploring solutions such as federated learning (where the AI comes to the data rather than sending data out) and robust encryption of any communication with AI services [50]. Access controls and audit trails are crucial—for example, logging every instance an LLM retrieves patient data and what it outputs. Some have even proposed blockchain technology to securely track all AI–EMR interactions as immutable records, which could help detect unauthorized data use or alterations [51]. Patient consent is another aspect: patients should be informed if an AI is involved in their care behind the scenes and how their data are used. Transparency builds trust; concealing AI involvement could backfire if errors occur. Ultimately, a multidisciplinary effort involving IT experts, legal/regulatory advisors, clinicians, and patient representatives is needed to ensure that introducing LLMs does not compromise the privacy and confidentiality at the core of the doctor–patient relationship.

### 5.2. Accuracy, hallucinations and patient safety

A well-documented phenomenon with generative AI is its tendency to sometimes produce fabricated or incorrect information with a confident tone, known as hallucination [52]. If a kidney patient asks an AI whether a certain herbal supplement will improve their kidney function and if the AI ‘invents’ a reassuring but false answer, the patient might act on that and delay proper care. Case reports of LLM confidently giving inaccurate medical advice or references have already arisen. For example, in its current form, LLM’s knowledge is derived largely from internet text, and it lacks a genuine understanding of the granular details of medicine. This might not be realized when extrapolating beyond evidence [53]. To mitigate this, any LLM-driven tool in nephrology must undergo rigorous validation and ideally constrain itself to well-supported information (possibly through the retrieval of trusted sources). From a safety standpoint, LLMs should not be given autonomous authority in clinical decision-making until we are certain that they meet high reliability standards. Some experts advocate that all patient-facing AI advice carry disclaimers such as ‘I am an AI assistant and not a substitute for professional medical advice’ and prompt users to double-check any recommendations, at least in this early phase. There is also work on refining LLM training (through reinforcement learning with human feedback and other alignment techniques) to reduce the incidence of harmful or false outputs [54,55]. Nevertheless, no AI is 100% error-free, so developing fail-safes (for example, an LLM that recognizes its own uncertainty and advises consulting a doctor in those cases) would be important for patient safety.

### 5.3. Bias and health equity

If the data contain biases—which healthcare data and literature often do—AI can perpetuate or even amplify those biases [56]. Nephrology has well-known disparities (for example, in access to transplantation among different ethnic groups or sexes). An LLM trained predominantly on English-language, Western datasets might perform poorly in understanding cultural contexts or nuances of care relevant to underrepresented populations [57]. Moreover, current LLMs operate primarily in a handful of languages and may not handle less common languages or dialects well. This language limitation could exacerbate health inequities, as patients or providers not fluent in the AI-supported languages might not benefit from it [58]. Bias can also manifest in AI content: for example, if most literature about a kidney disease is based on studies in men, an AI might inadvertently provide less accurate advice for women [59]. Ensuring diverse and representative training data is one approach to mitigate this, along with bias testing before deployment. The medical community is increasingly aware of these issues; many bioethicists stress that LLM development should involve stakeholders from marginalized communities to address their needs and concerns. The introduction of any AI into patient care should not worsen disparities but should ideally help reduce them. For example, if an LLM can be leveraged to provide high-quality kidney education to rural or resource-limited settings where specialists are scarce, that could be a large equalizer as long as the information is accurate and culturally appropriate.

### 5.4. Liability and professional ethics

Introducing LLMs in clinical pathways creates ambiguity in responsibility [60]. If a nephrologist uses an AI’s recommendation and a patient is harmed by that advice, who is at fault—the physician, the tool, the tool’s manufacturer? Currently, the standard view is that a clinician using any tool (AI or otherwise) is ultimately responsible for clinical decisions. This means that clinicians must not blindly follow AI output and should use it only as an adjunct to their own judgment. Some authors have argued that until LLMs meet reliability benchmarks accepted by the medical community, their role should remain assistive, with humans being firmly in charge. There are also emerging discussions about whether an AI system could be considered a medical device subject to regulatory approval (for example, by the FDA) [61]. If an AI is used in a manner that directly influences diagnosis or treatment (especially autonomously), regulators may require it to undergo evaluation for safety and efficacy similar to a drug or device. To date, no LLM has been FDA-approved for independent clinical decision-making. From an ethical standpoint, using AI in care also demands transparency and consent. Ethicists argue that patients have a right to know if their healthcare provider is consulting an AI model for their case or if the information given to them was generated by AI [62]. Legal frameworks need to catch up, potentially defining standards for AI in healthcare and establishing where liability lies (e.g., with the clinician, the institution, or the software developer) when things go wrong. Professional guidelines are also being drafted by groups such as the AMA and specialty societies to guide doctors in responsible AI use, emphasizing that AI should augment clinical practice and not replace professional responsibility.

### 5.5. Misinformation and ‘medical deep-fakes’

On a societal level, advanced text (and audio) generation raises the specter of misinformation [63]. This is an extension of the ‘deepfake’ problem (widely discussed with AI-generated images or videos) into the realm of medical advice. Patients might encounter AI-generated articles or social media posts with ­dangerously inaccurate information about home remedies for renal failure or conspiracy theories about dialysis, etc. The medical community and regulators will need to collaborate to combat such misinformation, through patient education on how to identify trustworthy information and possibly through technology that can verify authentic sources (for example, watermarking AI-generated content or using authentication systems for legitimate medical content). Another facet is plagiarism and intellectual property—AI can generate text that might inadvertently plagiarize sources or produce content that blurs authorship [64]. Academics must be cautious when using AI in writing papers or guidelines, ensuring credit and avoiding copyright infringement. Many of the issues discussed are surmountable with appropriate policy and technical solutions, but they require proactive effort. As one commentary put it, the medical community must ‘put guardrails in place’, even as we explore the exciting possibilities of ChatGPT-like systems in practice [65]. This means establishing accuracy standards, monitoring outcomes, and maintaining human judgment as the final say in patient care decisions.

## 6. Future directions

LLMs are rapidly becoming more capable, and their footprint in healthcare is expected to expand significantly in the coming years. In nephrology, we anticipate a progression from experimental use cases to more routine, albeit carefully supervised, integration of LLM-based tools in both clinical and educational workflows.

Current general LLMs might be augmented or fine-tuned on nephrology-specific data, for example, training on decades of nephrology literature, dialysis unit protocols, and kidney transplant databases. A ‘KidneyGPT’ could theoretically have deeper specialized knowledge, reducing the errors that come from knowledge gaps. However, obtaining and curating such specialty datasets (many of which may be behind paywalls or in registry silos) will be a challenge. Collaborative efforts between professional societies (such as the International Society of Nephrology) and AI developers could help create validated, up-to-date knowledge bases for AI. Moreover, future models will likely be larger and more powerful but also more efficient, possibly enabling onsite deployment (within a hospital’s secure server) rather than relying on cloud API calls. This could alleviate some privacy concerns and give institutions more control over model updates, ensuring that new medical evidence is incorporated regularly.

Research will continue to reduce hallucinations and improve the accuracy of LLM responses. Techniques such as retrieval augmentation (where the model retrieves relevant text from a medical database to ground its answers) and enhanced training with human feedback specific to medical Q&A are already underway [66,67]. Future iterations might approach a level where their answers can be trusted like a well-trained junior physician, at least for certain bounded tasks. Before that, benchmarking and validation in nephrology contexts will be critical. We may see formal evaluation of LLM performance on nephrology board examinations or in answering patients’ frequently asked questions. If an LLM consistently performs at a high level and improves over time with continued feedback learning, trust in its use will increase.

Beyond technical performance, the true impact of LLMs needs to be measured in clinical terms. Do patients who use a nephrology chatbot have better knowledge retention or treatment adherence? Can AI-assisted documentation free up 20% of a clinician’s time, and does that translate into better patient satisfaction or more preventive care delivered? Does an AI-augmented care model improve blood pressure control in CKD patients or reduce hospitalization rates? These empirical questions require prospective studies. Initial pilots might focus on process measures, such as time savings, the number of errors caught or introduced by AI, and user satisfaction scores. Indeed, some studies are already calling for more research on how chatbots could be safely incorporated for patient and clinician use [68]. By 2025 and beyond, we anticipate the emergence of clinical trials or at least implementation studies in which one group of clinics uses AI assistance and another does not to observe differences in efficiency and patient outcomes. Regulatory bodies may eventually mandate such evidence if AI tools are to be marketed for specific clinical uses.

Nephrology often overlaps with other specialties, such as cardiology for hypertension and endocrinology for diabetes. Future AI systems could be holistic, assisting in the management of comorbid conditions in an integrated way. For example, an AI care coach for a diabetic kidney disease patient would need to handle advice about blood sugar control and kidney protection. LLMs are well suited to such cross-domain counseling since they are not limited by medical specialty silos. This could encourage a more patient-centered rather than a specialty-centered approach in chronic disease care. We might see digital health platforms that embed LLMs to provide a one-stop resource for patients managing multiple conditions, with appropriate input from their array of human providers.

**Figure 2.**
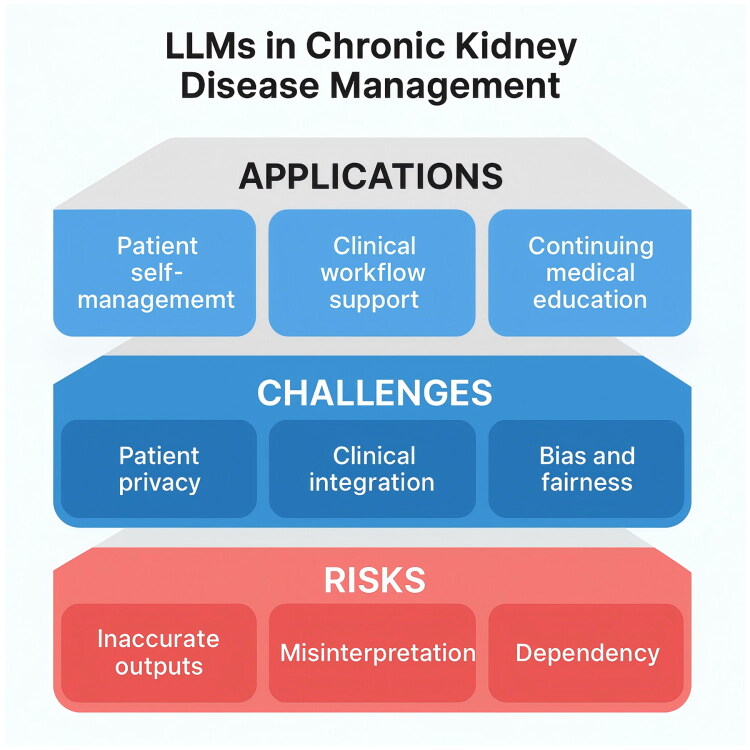
Integrative 3D map of applications, challenges, and risks of LLMs in CKD management. This figure presents a structured overview of the principal applications, core challenges, and potential risks associated with the integration of large language models (LLMs) in chronic kidney disease management.

As the role of LLMs solidifies, nephrology training may evolve to include AI literacy—teaching clinicians how to effectively use and supervise AI tools [69]. Just as today’s medical trainees learn to use clinical decision support systems and drug interaction checkers, future trainees might be taught how to query AI, how to critically appraise AI outputs, and how to correct AI errors. New professional roles might emerge, such as ‘clinical AI auditors’ or ‘medical AI navigators,’ which ensure that the deployment of an LLM in a clinic functions as intended and that end-users are comfortable with it. Guidelines and best practices will likely be published for various use cases (e.g., ‘Best practices for using AI chatbots in patient education for dialysis’). Additionally, involving patients in codesigning these AI services will be important – their feedback on what explanations are clear or what tone is comforting should shape how the LLM interacts. This ability could enhance patient engagement and trust in the healthcare system. Conversely, missteps (like an AI giving a serious diagnosis in an insensitive manner) could cause backlash, which we must strive to avoid through ethical design and thorough testing. To provide a comprehensive overview of the current landscape and future priorities for LLMs in chronic kidney disease management, we present an integrative conceptual framework (Figure 2) that visually summarizes the key applications, primary challenges, and potential risks associated with these technologies.

## 7. Conclusion

LLMs represent transformative technology that is poised to impact nephrology in multiple dimensions, from how patients learn about their kidney health to how clinicians document and deliver care and how the next generation of nephrologists are educated. Nephrology, with its rich data and pressing need for patient education, stands to be at the forefront of this AI-driven transformation in healthcare. By addressing the current limitations and building robust frameworks for human–AI collaboration, LLMs could become an invaluable adjunct in nephrology. Through prudent stewardship, LLM technology can be harnessed to improve clinical outcomes and patient experiences, marking a new era of digitally enhanced kidney care.
